# The efficacy and safety in radioactive iodine refractory thyroid cancer patients treated with sorafenib

**DOI:** 10.3389/fendo.2023.1200932

**Published:** 2023-07-18

**Authors:** Yuanna Ling, Xiaoli Xiong, Jiaxin Luo, Quanliang Zou, Pan Chen, Liqin Pan, Man Long, Huijuan Feng, Wei Ouyang

**Affiliations:** ^1^ Department of Nuclear Medicine, Zhujiang Hospital, Southern Medical University, Guangzhou, Guangdong, China; ^2^ Department of Ultrasound Medicine, Zhujiang Hospital, Southern Medical University, Guangzhou, Guangdong, China

**Keywords:** differentiated thyroid cancer, sorafenib, radioactive iodine refractory, real-world clinical study, efficacy and safety

## Abstract

**Background:**

Sorafenib included in Chinese medical insurance is the earliest targeted drug for radioactive iodine refractory differentiated thyroid cancer (RR-DTC). This study is to further demonstrate the clinical efficacy and safety of sorafenib used in Zhujiang Hospital of Southern Medical University.

**Methods:**

RR-DTC patients treated at our Department of Nuclear Medicine in Zhujiang Hospital of Southern Medical University (October 2017–May 2020) were retrospectively analyzed. Treatment effects, progression-free survival (PFS), and adverse effects (AEs) during medication were evaluated.

**Results:**

Of the 31 patients included, 26 patients were evaluated for efficacy with a median follow-up time of 17.5 months (4.0–51.0 months). The disease control rate (DCR) was 57.7% (*n* = 15) and the objective response rate (ORR) was 26.9% (*n* = 7). Most patients with disease control had thyroglobulin decreases of more than 60% (*p* = 0.004), ORRs were favorable in patients with lung metastasis and lung-only metastasis (*p* = 0.010 and 0.001, respectively). The PFS of the 26 patients analyzed was 16.5 months (95%CI: 14.41 –23.90 months). In the subgroup analysis, female, patients with lung-only metastasis, hand-foot skin syndrome (HFS), and thyroglobulin response ≥ 60% observed longer PFS (*p* = 0.038, 0.045, 0.035, and 0.000, respectively), while patients with bone metastasis had lower PFS (*p* = 0.035). The most common toxicity profile was HFS (93.5%), followed by diarrhea (83.9%), alopecia (74.2%). All the side effects were mainly grade 1–2. Grade 3–4 adverse reactions were more common in diarrhea and HFS.

**Conclusions:**

Sorafenib has promising efficacy in RR-DTC, especially in patients with lung metastasis and lung-only metastasis. The AEs of sorafenib were generally mild, and the main AE was HFS.

## Introduction

Thyroid cancer is a common endocrine tumor, ranking seventh among all cancer types reported in China in 2022, with the fastest-growing incidence (1, 2). Differentiated thyroid cancer (DTC) is accounted for 95% of all histological types of thyroid cancer, including papillary thyroid cancer (PTC), follicular thyroid cancer (FTC), and Hürthle cell cancer (3). In general, thyroid cancer presents an excellent prognosis with standard management including surgery, radioactive iodine (RAI) treatment, and thyroid-stimulating hormone suppression therapy (4–6). However, about 20% and 10% of DTC patients are at risk of local recurrence and distant metastases respectively, and two-thirds of them lose the ability of RAI uptake initially or gradually, which was known as radioactive iodine refractory differentiated thyroid cancer (RR-DTC) (7). As limited treatment options result in poor prognosis, the 10-year survival rate is less than 10% in RR-DTC patients (8, 9).

Sorafenib, approved by the U.S. Food and Drug Administration and the State Food and Drug Administration of China for the treatment of RR-DTC, is an oral tyrosine kinase inhibitor (TKI) inhibiting vascular endothelial growth factor receptors (VEGFRs), RAF, RET, and platelet-derived growth factor receptor beta signaling (10–12). Compared with the placebo, sorafenib improved the progression-free survival (PFS) of RR-DTC patients showing efficacy and safety in DECISION trial (13). Real-world studies not only can reflect the effects of drugs and provide more important health condition information about patients in their daily life but also provide a more scientific basis guiding physicians’ clinical decision. Further demonstration of the clinical efficacy and safety of sorafenib used in different populations, centers, and age groups is essential.

Although many real-world studies on the treatment of RR-DTC with sorafenib have been reported (14–19), there are a few studies on the efficacy and adverse effects (AEs) of sorafenib at a standard dose of 400mg twice daily in Zhujiang Hospital of Southern Medical University. Our research not only analyzed the efficacy and AEs of sorafenib at a standard dose of 400mg twice daily for the treatment of RR-DTC patients at our Department of Nuclear Medicine from October 2017 to May 2020 but also summarized the real-world studies on sorafenib reported to date in the Discussion section.

## Material and methods

Patients diagnosed with RR-DTC at our Department of Nuclear Medicine in Zhujiang Hospital of Southern Medical University from October 2017 to May 2020 were retrospectively analyzed. All patients were given sorafenib at the standard dose of 400mg, twice a day, and then the dose was adjusted according to the patient’s tolerance to side effects. Patients who received other treatments while taking sorafenib or were interrupted in follow-up were excluded. RR-DTC was defined as follows (if one of the followings is satisfied): 1) at least one target lesion without RAI uptake (never or ever); 2) progression of a target lesion despite significant RAI concentration; 3) cumulative RAI dose ≥ 22.3 GBq. Imaging examinations were performed every 3 to 6 months to assess efficacy, AEs were assessed monthly. The Response Evaluation Criteria in Solid Tumors version 1.1 was used to evaluate treatment efficacy (20): disease control rate (DCR) referred to complete response (CR) plus partial response (PR) plus stable disease (SD), objective response rate (ORR) was CR plus PR, and progression-free survival (PFS) was defined as the time from initiation of sorafenib to progression or death. Common Terminology Criteria for Adverse Events Version 5.0 was used to evaluate AEs and guide patients to adjust their medication in response to side effects: if patients had grade 1–2 AEs, the dose could be appropriately adjusted according to the patient’s ability to tolerate the AEs, if the patients had grade 3 or above AEs, the medication would be suspended until the AEs returned to grade 1–2. (Available from: https://ctep.cancer.gov/protocolDevelopment/electronic_applications/ctc.htm#ctc_50. Accessed 15 May 2020).

Baseline characteristics of patients were described: quantitative data were reported as mean or median, and categorical variables as numbers with percentages. Waterfall plots were used to present the best tumor response. The PFS was calculated using the Kaplan–Meier method, and a log-rank test was performed to compare the difference in the PFS of variables. Cox proportional hazard model was used to estimate prognostic factors associated with PFS. All data were analyzed using SPSS version 26.0, and a p-value < 0.05 was considered statistically significant.

## Results

### Baseline characteristics

**Table 1** summarizes the clinicopathological profiles of 31 retrospective patients with a median age of 58 years (18–79 years) when starting sorafenib, most of whom had PTC (61.3%). Twenty-four (77.4%) patients had distant metastases, half of whom had bone metastases and 16 had lung metastases. Of the 31 patients, five were excluded for the following reasons: three had only one radiological examination, one was absent of any measurable target lesion, and one underwent surgery during sorafenib therapy. The other 26 patients were eligible for radiological response assessment with a median follow-up of 17.5 months (4.0–51.0 months). AEs during sorafenib administration were recorded in 31 patients with a median follow-up of 18 months (2.0–51.0 months).

**Table 1:** Baseline clinicopathologic characteristics of patients treated with sorafenib.

	Median (range) or *N* (%)
Age (years)	58 (18–79)
≥ 55	19 (61.3)
< 55	12 (38.7)
Gender	
Male	12 (38.7)
Female	19 (61.3)
Histologic types	
FTC	10 (32.3)
PTC	19 (61.3)
FTC + PTC	2 (6.4)
RAI uptake	
Yes	20 (64.5)
No	11 (35.5)
Distant metastases	
Yes	24 (77.4)
No	7 (22.6)
Metastasis sites	
Lung	16 (66.7)
Lung-only	7 (29.2)
Bone	12 (50.0)
Pleural	1 (4.2)
Target lesions	
Lung	14 (45.2)
Bone	9 (29.0)
lymph node	1 (3.2)
Neck	7 (22.6)
Cumulative RAI dose (GBq)	11.7 (0.1–58.8)
Definitions of RAI refractoriness	
At least one target lesion without RAI uptake	10 (32.3)
RAI uptake,but progressive target lesions	17 (54.8)
Disease progression even with RAI therapy or cumulative RAI dose ≥ 22.3GBq	4 (12.9)

FTC, follicular thyroid cancer; PTC, papillary thyroid cancer; RAI, radioactive iodine.

### Efficacy of sorafenib

**Figure 1:**
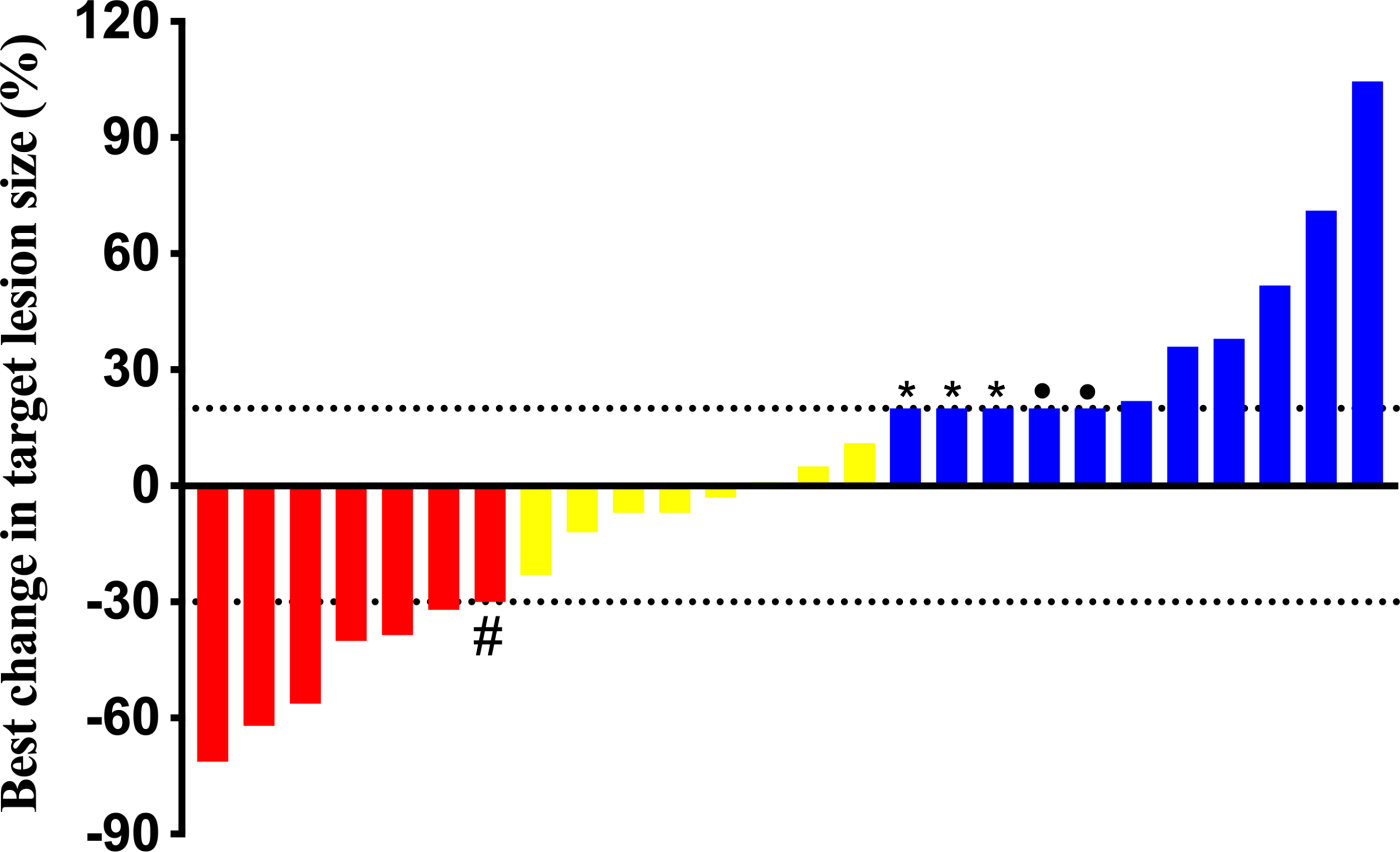
Best change in target lesions of 26 patients. #: This patient’s chest computed tomography scan showed micro-nodules scattered throughout the lungs which couldn’t be determined by the Response Evaluation Criteria in Solid Tumors version 1.1, but we found a significant reduction in his lung micro-nodules and a 91.4% reduction in thyroglobulin after follow-up, thus we considered a partial response. *: All three patients were found with new lesions. •:The two patients were died to pulmonary infection and bone related events, respectively.

As presented in **Table 2**, no one achieved CR, 26.9% (*n* = 7) of patients achieved PR and all exceeded 12 months, and 30.7% (*n* = 8) had SD. The DCR was 57.7% (*n* = 15), with 14 cases controlled for ≥ 12 months, nine cases for ≥ 18 months, and three cases for ≥ 24 months. Eleven (42.3%) had documented disease progression at the end of follow-up, three of whom recorded new metastases rather than enlargement of target lesions. **Figure 1** demonstrates the best changes in target lesions.

**Table 2:** Treatment efficacy of patients treated with sorafenib.

	*N* (%)
Disease response	
CR	0
PR	7 (26.9%)
SD	8 (30.7%)
Disease control (CR+PR+SD)	15 (57.7%)
Disease progression	11 (42.3%)
Disease control duration	
≤ 6months	1 (3.8%)
6–12 months	0
12–18 months	5 (19.2%)
≥ 18months	9 (34.6%)
Objective response ≥ 12 months	7 (26.9%)

CR, complete response; PR, partial response; SD, stable disease.

As illustrated in **Table 3**, there were no significant differences in DCRs and ORRs according to age, gender, histologic types, RAI uptake, distant metastases, bone metastasis, previous treatment modality, residual lesions, and hand-foot skin syndrome (HFS). Notably, DCRs were superior in patients with a reduction of ≥ 60% in thyroglobulin (Tg) (*p* = 0.004). ORRs were favorable in patients with lung metastasis and lung-only metastasis (*p* = 0.010 and 0.001, respectively).

**Table 3:** The association between prognostic features and response to sorafenib.

Variables	DCR, %	*p*-value	ORR, %	*p*-value
Age (years)		1.000		0.661
≥ 55	58.8		23.5	
< 55	55.6		33.3	
Gender		0.233		1.000
Male	41.7		25.0	
Female	71.4		28.6	
Histologic types		1.000		0.661
FTC	55.6		33.3	
PTC	58.8		23.5	
RAI uptake		1.000		0.665
Yes	57.1		21.4	
No	58.3		33.3	
Distant metastases		1.000		0.134
Yes	57.9		36.8	
No	57.1		0	
Lung metastasis		1.000		**0.010**
Yes	60.0		46.7	
No	54.5		0	
Lung-only metastasis		0.084		**0.001**
Yes	87.5		75.0	
No	44.4		5.6	
Bone metastasis		0.228		0.190
Yes	40.0		10.0	
No	68.8		37.5	
Previous therapies		1.000		1.000
RAI-only therapy	58.3		25.0	
Other therapy	57.1		28.6	
Residual lesions		1.000		0.629
Yes	57.1		14.3	
No	57.9		31.6	
Hand-foot syndrome		0.423		1.000
Yes	60.0		28.0	
No	0		0	
Thyroglobulin response		**0.004**		0.081
≥ 60%	85.7		42.9	
< 60%	25.0		8.3	
Medication status		0.683		0.635
Reduction/discontinuation	61.1		22.2	
Standard dose	50.0		37.5	

DCR, disease control rate; ORR, objective response rate; FTC, follicular thyroid cancer; PTC, papillary thyroid cancer; RAI, radioactive iodine.

The bold values are statistically significant.

**Figure 2:**
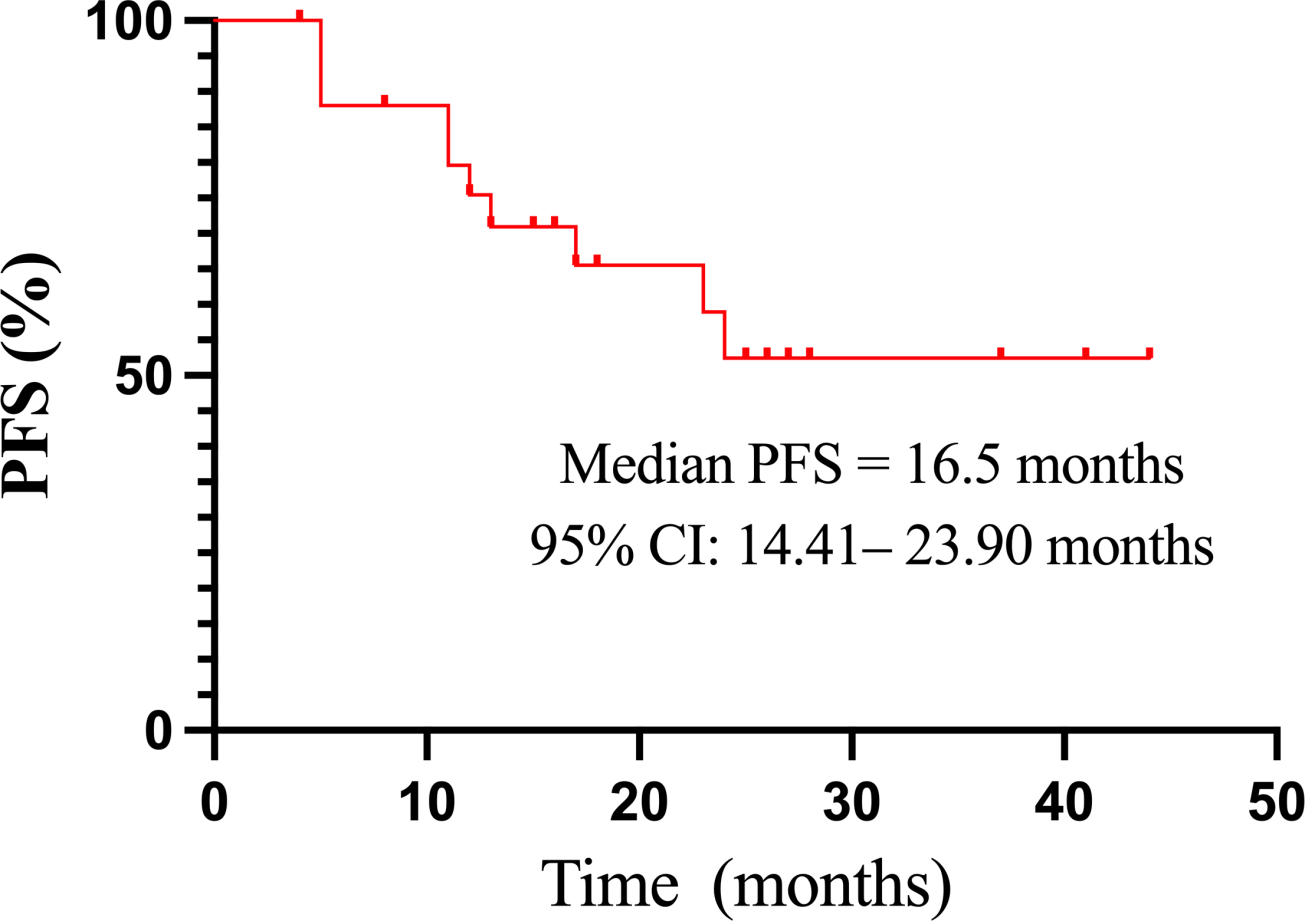
The Kaplan–Meier curve of PFS in 26 patients treated with sorafenib. CI, confidence interval; PFS, progression-free survival.

**Figure 3:**
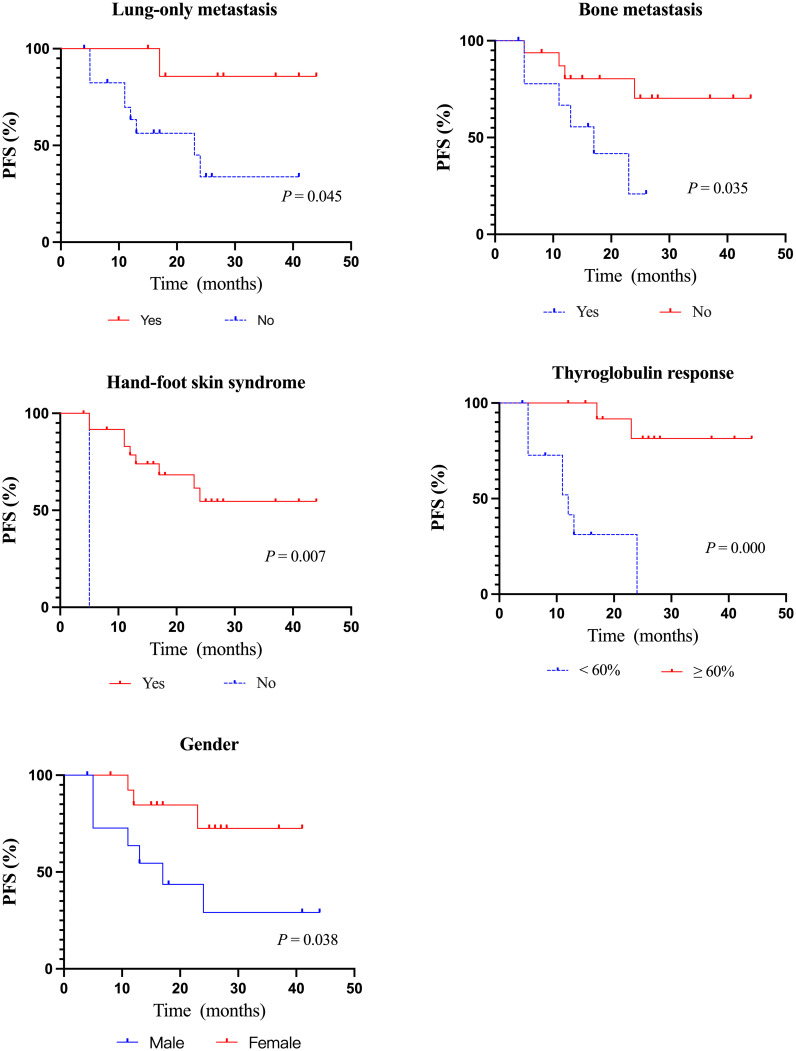
Kaplan–Meier curves of PFS according to clinicopathological features. PFS, progression-free survival.

The median PFS of all 26 evaluable patients was 16.5 months (95%CI: 14.41–23.90 months) (**Figure 2**). There were no significant differences in PFS concerning age, histologic type, RAI uptake, distant metastases, lung metastasis, previous treatment modality, and residual lesions as indicated in **Table 4**. PFS was better in female and patients with lung-only metastasis, HFS, and Tg response ≥ 60% (*p* = 0.038, 0.045, 0.007, and 0.000, respectively); however, it was worse in patients with bone metastasis (*p* = 0.035). Nineteen patients with distant metastasis were divided into groups with and without bone metastasis, and survival analysis showed a worse PFS with the former (*p* = 0.062) (**Table 4**; **Figure 3**).

**Table 4:** Univariate analyses of potential prognostic factors associated with the PFS of patients treated with sorafenib.

Variables	*N* (%)	PFS, months	*p*-value
Age (years)			0.744
≥ 55	17 (65.3)	29.607	
**<** 55	9 (34.6)	30.130	
Gender			**0.038**
Male	12 (46.2)	21.691	
Female	14 (53.8)	34.286	
Histologic types			0.776
FTC	9 (34.6)	28.075	
PTC	17 (65.4)	28.995	
RAI uptake			0.644
Yes	14 (53.8)	29.587	
No	12 (46.2)	28.483	
Distant metastases			0.621
Yes	19 (73.1)	28.900	
No	7 (26.9)	31.000	
Lung metastasis			0.696
Yes	15 (57.7)	29.214	
No	11 (42.3)	29.156	
Lung-only metastasis			**0.045**
Yes	8 (30.8)	39.875	
No	18 (69.2)	23.045	
Bone metastasis			**0.035**
Yes	10 (38.5)	16.347	
No	16 (61.5)	35.201	
Previous therapies			0.655
RAI therapy	12 (46.2)	29.917	
No-RAI therapy	14 (53.8)	28.573	
Residual lesions			0.894
Yes	7 (26.9)	26.667	
No	19 (73.1)	30.080	
Hand-foot skin syndrome			**0.007**
Yes	25 (96.2)	30.705	
No	1 (3.8)	5.000	
Disease control			**0.000**
PR + SD	15 (57.7)	–	
Disease progression	11 (42.3)	–	
Objective response			**0.017**
PR + CR	7 (26.9)	–	
SD + disease progression	19 (73.1)	–	
Thyroglobulin response			**0.000**
≥ 60%	14 (53.8)	39.611	
< 60%	12 (46.2)	13.727	
Medication status			0.396
Reduction/discontinuation	18 (69.2)	31.688	
Standard dose	8 (30.8)	18.406	

PFS, progression-free survival; FTC, follicular thyroid cancer; PTC, papillary thyroid cancer; RAI, radioactive iodine; CR, complete response; PR, partial response; SD, stable disease;–, no data; Distant metastases^1^, Distant metastasis of all patients; Distant metastases^2^.

The bold values are statistically significant.

–, not reached.

The dose was adjusted according to the patient’s tolerance to side effects. Five patients discontinued the drug for AEs and resumed the drug after the AEs were alleviated, with the longest interval of 6 months. The others’ minimum maintenance dose was 200mg daily. There were no significant differences in DCR, ORR, and PFS between patients with dose reduction or withdrawal of sorafenib and patients with continuous treatment (**Tables 3**, **4**).

### Safety and AEs

AEs were documented in all patients as revealed in **Table 5**. The most frequent AE of any grade was HFS (93.5%), followed by diarrhea (83.9%), alopecia (74.2%), and so on. These AEs were mainly grades 1–2. Similarly, diarrhea (16.1%) and HFS (9.7%) were the most prevalent among grades 3–4. Moreover, a few uncommon AEs such as weight loss (*n* = 1), myodynia (*n* = 4), and thrombocytopenia (*n* = 4) were reported. Most side effects were persistent, especially HFS, diarrhea, and alopecia, with the longest lasting 44 months. The median duration of HFS was 18 months (range: 0.5–44 months), the median duration of diarrhea was 16.5 months (range: 1–44 months), and the median duration of alopecia was 12.0 months (range: 2–44 months). Grade 3-4 AEs were common with HFS, diarrhea and abnormal liver function for a maximum duration of 38 months. Nineteen (61.3%) patients had drug reductions due to AEs (some patients had more than one AEs: HFS, *n* = 19; diarrhea, *n* = 5; abnormal liver function, *n* = 1; proteinuria, *n* = 1).

**Table 5:** Adverse events occurring in patients treated with sorafenib.

	Grades 1–2	Grades 3–4	Any grade
Adverse events	*N* (%)	*N* (%)	*N* (%)
Hand-foot skin syndrome	26 (83.9)	3 (9.7)	29 (93.5)
Diarrhea	21 (67.7)	5 (16.1)	26 (83.9)
Alopecia	23 (74.2)	0	23 (74.2)
Abnormal liver function	15(48.4)	2 (6.5)	17 (54.8)
Generalized weakness	17 (54.8)	0	17 (54.8)
Hypertension	13 (41.9)	2 (6.5)	15 (48.4)
Proteinuria	10 (32.3)	2 (6.5)	12 (38.7)
Hypocalcemia	9 (29.0)	1 (3.2)	10 (32.3)
Mucositis	8 (25.8)	0	8 (25.8)
Nausea and vomiting	6 (19.4)	1 (3.2)	7 (22.6)
Thrombocytopenia	4 (12.9)	0	4 (12.9)
Myodynia	4 (12.9)	0	4 (12.9)
Weight loss	1 (3.2)	0	1 (3.2)

### Typical cases

**Figure 4:**
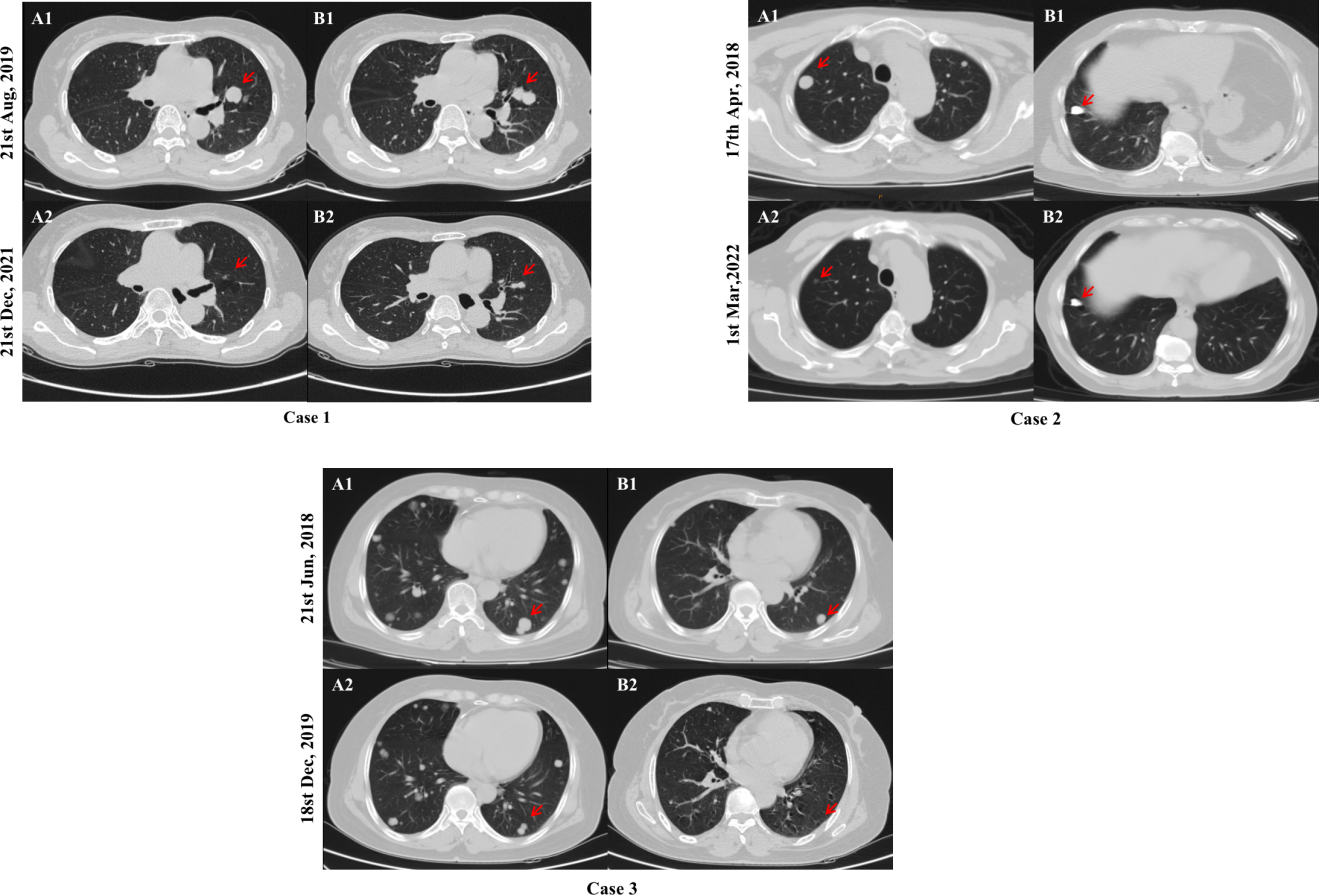
Comparison of chest computed tomography scans of typical cases. After 28, 46, and 17 months of sorafenib treatment, a significant reduction in the size of target lesions was observed in cases 1, 2, and 3, respectively. Chest computed tomography scans at baseline (A1 and B1) and the end of follow-up (A2 and B2) are shown above. The red arrows indicates the location of the target lesions at baseline and the last follow-up.

Patients 1, 2, and 3 were diagnosed with RR-DTC on 08/09/2018, 06/20/2018, and 08/30/2019, respectively, and started taking sorafenib using 400 mg twice daily, followed by regular follow-up. Only lung metastases were found in all of them, and all target lesions were lung metastases. As depicted in **Figure 4**, at the end of the follow-up, their chest computed tomography scans (12/21/2021, 03/01/2022, and 12/18/2019, respectively) showed that their target lesions significantly reduced by ≥ 30% or even disappeared compared with the baseline data, hence they were evaluated as PR.

## Discussion

As shown in **Table 6**, we summarize the real-world studies reported so far and the DECISION trial (13, 14, 16, 18, 19). The DCR of our study was 57.7%, similar to most of the above studies. However, the ORR in our study was slightly improved to 26.9% compared with those reported by Benekli et al., Massicotte et al., Kim et al., and Cheng et al. (14.0%,15.0%, 10.3%, and 21.7%, respectively). Furthermore, the median PFS in our study was 16.5 months, longer than those of DECISION, Kim et al., and Massicotte et al. (10.8, 9.7, and 7.2 months, respectively) (13, 14, 18), which may be due to the different tumor burdens of patients included such as those with advanced disease or distant metastases (14, 16, 18). However, the median PFS of the studies by Cheng et al. and Benekli et al. (17.6 and 21.3 months, respectively) was longer than ours, and a longer follow-up was found in the study of Cheng et al. than ours (median follow-up: 25.1 months versus 17.5 months) possibly resulting in longer PFS. Overall, both the clinical trial and real-world studies have demonstrated considerable efficacy in treating RR-DTC with sorafenib.

**Table 6:** Reported real-world studies and the DECISION trial.

Authors	Year	Study design	Patients included	Sample sizes	Follow-up time (months)	DCR (%)	ORR (%)	PFS (months)	AEs (%)
Brose et al.	2014	Phase III RCT multicenter	96.4% metastatic or locally advanced RR-DTC	207	16.2	54.1 (≥ 6 months)	12.2	10.8	98.6
Benekli et al.	2014	single center prospective	RR-DTC	14	21.3	56.9	14.0	21.3	NM
Massicotte et al.	2014	single center prospective	Metastatic or locally advanced RR-DTC	39	NM	92.0	15.0	7.2	82.1
Kim et al.	2019	retrospective multicenter	RR-DTC	85	9.1	67.0	10.3	9.7	95.0
Cheng et al.	2020	single center prospective	RR-DTC	72	17.6	73.3	21.7	17.6	73.6

ORR, objective response rate; DCR, disease control rate; PFS, progression-free survival; AEs, adverse events; RCT, randomized controlled trial; NM, not mentioned; DTC, differentiated thyroid cancer; RR-DTC, radioactive iodine refractory differentiated thyroid cancer.

Studies have shown that soft tissue metastases such as lymph nodes, pleura, and pulmonary lesions presented better outcomes (16, 18, 21). Similarly, our study discovered that Sorafenib is more effective in lung metastases, all patients with PR were lung metastasis and patients with lung-only metastasis generally had a better prognosis such as typical cases shown in **Figure 4**, while those with bone metastasis presented a worse prognosis (**Table 4**). Besides, in the subgroup analysis of the relationship between PFS and clinicopathological features, as shown in **Table 4**, PFS was significantly better in patients with lung-only metastases and significantly worse in patients with bone metastases, in the subgroup analysis of PFS between the presence and absence of bone metastasis groups, the bone metastasis group tended to have worse PFS. We believe that the reasons are as follows: VEGF stimulates endothelial cell proliferation and tumor angiogenesis, playing an important role in the development and progression of thyroid cancer, and its expression level correlates with advanced disease (22, 23). Sorafenib is a multitargeted TKI whose core mechanism is to block the activity of VEGFR-2 and VEGFR-3 to inhibit tumor angiogenesis and cell proliferation. VEGFR is mainly expressed in soft tissues such as heart, lung, kidney, and skeletal muscle (24). Therefore, we estimate that lower VEGFR signal transduction in bone may be responsible for the poor efficacy of bone metastases in line with the study of Cheng et al. (16). Beyond that, pathophysiological processes in the bone microenvironment promote the rapid progression of bone lesions, and complex bone composition and structure make it difficult to be targeted, which leads to the rapid development of bone metastases (25–27). Therefore, patients with lung metastases respond relatively well to sorafenib treatment. For bone metastasis, anti-angiogenic therapy alone may be less effective, and a multimodal approach including local and systemic therapies should be considered. However, in this cohort, patients with bone metastasis just tended to have worse PFS, and the *p* value was still greater than 0.05, which still needs more data to further confirm.

In our cohort, male had worse PFS than female (**Table 4**; **Figure 3**). There are also various opinions on the relationship between gender and thyroid cancer prognosis. Some studies have shown that the prognosis of thyroid cancer in male patients is worse than that in female patients (28, 29), it may be related to the higher risk of radiation exposure and aggressive characteristics of male (28). However, there is no relevant study on the relationship between the efficacy of targeted drugs in RR-DTC treatment and gender, and we will further explore in our follow-up study.

Serum Tg is a well-recognized prognostic indicator for the presence of metastases or occult lesions after total thyroidectomy (6, 30, 31). However, RR-DTC lesions have various degrees of dedifferentiation, resulting in the loss of the ability to produce Tg; thus, it is controversial to use Tg to evaluate curative effects (32, 33). Our subgroup analysis revealed that patients with ≥ 60% decrease in Tg had longer PFS and better DCRs. Besides, patients in PR had a significant diminishment of the best change in Tg (more than a 90% decrease in six cases and a 58.2% decrease in one case). Kim et al. also found that a ≥ 60% decrease in Tg was associated with longer PFS and DCRs (14), and a similar conclusion was expounded by Cheng et al. (16). In contrast, Benekli et al. emphasized that Tg levels had no significance in prognosis or predicting response (19), and decreased or no change in Tg was observed in patients with advanced thyroid cancer treated with other TKIs (21, 34, 35). Whether the measurement of Tg can monitor therapeutic effects remains to be determined, but dynamic monitoring of changes in Tg such as the degree of Tg diminishment and Tg-doubling time can evaluate the efficacy of postoperative patients with thyroid cancer to a certain extent (31).

In our study, dose reduction and drug withdrawal occurred in 42.3% (*n* = 18) of patients due to AEs, mainly because of HFS, and a lower rate of AEs was found compared with the DECISION trial (93.1% versus 98.6%) (13), but a lower rate of AEs was found in the study of Cheng et al. (73.6%) than ours; therefore, a higher dose may result in a higher rate of AEs (16). Since common AEs were HFS, diarrhea, alopecia, abnormal liver function, generalized weakness, and hypertension, clinicians should closely monitor patients’ skin condition, blood pressure, and general situation when using sorafenib to avoid dose reduction or discontinuation.

It has been confirmed that patients treated with TKIs are at high risk of HFS (36). Virtually all patients had HFS (93.5%), most of them were grades 1–2, and only three were grades 3 and above. Moreover, the occurrence of HFS was correlated with better PFS in our analysis. Many studies have confirmed that HFS is associated with a higher cumulative sorafenib dose and with anti-VEGF and anti-VEGFR activities (17, 37). Since anti-angiogenesis is one of the main mechanisms of sorafenib in treating RR-DTC, HFS could be a significant therapeutic index during the administration of TKIs (38, 39). Thus, clinicians could estimate whether TKIs are starting to work based on the appearance of HFS.

As the sample size included in our study was not large and the median follow-up was only 17.5 months, no factors affecting PFS were identified in multivariate analysis. We will continue to follow up with patients for PFS and include more patients to analyze the factors influencing PFS.

## Data availability statement

The raw data supporting the conclusions of this article will be made available by the authors, without undue reservation.

## Ethics statement

The studies involving human participants were reviewed and approved by Medical Ethics Committee of Zhujiang Hospital of Southern Medical University. Written informed consent for participation was not required for this study in accordance with the national legislation and the institutional requirements.

## Author contributions

YL, WO and HF designed the study. YL and XX collected and analyzed the data and wrote the manuscript. YL, XX, and JL checked and corrected the data and manuscript. QZ, PC, LP, and ML participated in measuring and collecting the data. WO and HF reviewed and edited the manuscript and approved the final draft. All authors contributed to the article and approved the submitted version.
